# Associations of deliberate self-harm with loneliness, self-rated health and life satisfaction in adolescence: Northern Finland Birth Cohort 1986 Study

**DOI:** 10.3402/ijch.v72i0.21085

**Published:** 2013-08-05

**Authors:** Anna Reetta Rönkä, Anja Taanila, Markku Koiranen, Vappu Sunnari, Arja Rautio

**Affiliations:** 1 Women's and Gender Studies, Faculty of Education, University of Oulu, Finland; 2 Institute of Health Sciences, University of Oulu, Finland; 3 Primary Health Care Unit, University Hospital of Oulu, Oulu, Finland; 4 Thule Institute, University of Oulu, Finland

**Keywords:** deliberate self-harm, loneliness, adolescents, gender, Northern Finland

## Abstract

**Background:**

Deliberate self-harm (DSH) is an act with a non-fatal outcome in which an individual initiates a behavior, such as self-cutting or burning, with the intention of inflicting harm on his or her self. Interpersonal difficulties have been shown to be a risk factor for DSH, but the association between subjective experience of loneliness and DSH have rarely been examined.

**Objective:**

To examine the frequency of DSH or its ideation and loneliness among 16-year-olds to determine if associations exist between DSH and loneliness, loneliness-related factors, self-rated health and satisfaction with life.

**Design:**

The study population (n=7,014) was taken from Northern Finland Birth Cohort 1986 (N=9,432). Cross-tabulations were used to describe the frequency of DSH by factors selected by gender. Logistic regression analysis was used to describe the association between DSH and loneliness and other selected factors.

**Results:**

Nearly 8.7% (n=608) of adolescents reported DSH often/sometimes during the preceding 6 months, with girls (n=488, 13.4%) reporting DSH almost 4 times than that of boys (n=120, 3.6%). Nearly 3.2% of the adolescents (girls: n=149, 4.1%; boys: n=72, 2.2%) expressed that the statement *I feel lonely* was very/often true, and 26.4% (girls: n=1,265, 34.8%; boys: n=585, 17.4%) expressed that the statement was somewhat/sometimes true. Logistic regression showed that those who reported to be very/often lonely (girls: odds ratio (OR) 4.1; boys: OR 3.2), somewhat/sometimes lonely (girls: OR 2.4; boys: OR 2.4) were dissatisfied with life (girls: OR 3.3; boys: OR 3.3), felt unliked (girls: OR 2.2; boys: OR 6.0) and had moderate self-rated health (girls: OR 2.0; boys: OR 1.7), were more likely to report DSH than those without these feelings.

**Conclusion:**

The results show that loneliness is associated with DSH, and that loneliness should be considered as a risk for individual health and well-being.

Suicide is one of the leading causes of death among adolescents in the Western world (1). In Finland, suicide rates of children and adolescents are among the highest in the world (2). Every year, more than 100 Finnish children or adolescents aged 10–24 commit suicide (3). The suicide rates are higher among males, while adolescent females report more deliberate self-harm (DSH), suicide ideation and attempts (2,4). However, the suicide rate for adolescent girls in Finland has steadily increased over the last few years (2).

In our study, we examine DSH and its association with loneliness and related factors, self-rated health and life satisfaction among Finnish adolescents. DSH is an act with a non-fatal outcome in which an individual initiates a behavior (such as self-cutting, burning, ingesting excess medicines/drugs, ingesting a non-ingestible substance/object, jumping from a height) with an intent to harm his or her self (5). DSH is common in adolescence; the reported mean lifetime prevalence of DSH is 13.2% (6) and prevalence during the preceding 12 months ranges from 6 to 26% (4,6). Documented motives for DSH include wanting to get relief from distress, to escape from (difficult) situations and to show how desperate one is feeling; it is a cry for help (7). Unnoticed or untreated DSH may precede suicide (1).

Risk factors for DSH, suicidal ideation and completed suicides are often similar and include depression (8,9), excessive alcohol and drug use (1), physical and sexual abuse (8) and interpersonal difficulties, such as having troubled or poor relationships with peers and family (1,5). Therefore, the social domain, including experiences of loneliness, seems to be closely related to DSH (10). However, very little research has been conducted on the associations between DSH and subjective experiences of loneliness.

Loneliness is a subjective, cross-cultural experience and part of the human condition that negatively affects the overall well-being. It also carries a health risk (11). In our study, loneliness is regarded as a negative, involuntary experience, which is in line with the large body of research on loneliness (11). Adolescent loneliness has been related to many negative mental and physical health and well-being factors, such as depression, high social anxiety (11), low self-esteem (12), victimisation of bullying (13) and poor self-rated health (14). Among young adults, chronic feelings of loneliness have been associated with disturbances in sleep and higher blood pressure and cardiovascular functioning (15). Loneliness is very common in adolescence (age: between 12 and 22 years); 20–50% of all adolescents experience some degree of loneliness (11,16).

Few studies have examined the relationship of subjective experience of loneliness to DSH before (17). Furthermore, very few population-based cohort studies have examined DSH (9,18). Small sample sizes are typical of the Northern communities when examining suicidal behavior and, overall, many earlier studies of adolescent suicidal behavior have been conducted on psychiatric samples or on hospital admissions. These can result in research designs with low statistical power (19), and these kinds of data might distort or underestimate the prevalence of suicidal behavior, including DSH. Therefore, findings from non-clinical populations will be more representative of adolescents (6). Furthermore, cohort data have been used to examine experiences of loneliness among the adult and the elderly in Finland (20) and elsewhere (21), but the experiences of loneliness in adolescents have rarely been examined in large population-based cohort studies. Two different population-based cohorts, 20 years apart, have been collected from Northern Finland, entitled *Northern Finland Birth Cohort (NFBC) 1966 and NFBC 1986*. One purpose of these cohorts is to follow societal changes and to examine the changes in the psychosocial well-being of the cohort members in the same area. These cohorts are the only Finnish data, which have examined longitudinally and this rigorously the life path of individuals in Northern Finland, both physically and psychically. Our data offer a unique opportunity to understand the association between DSH and loneliness, and this study is the first to examine the theme of loneliness in the NFBC 1986 data.

The rates for suicidal behavior are different for boys and girls (5), and different genders might experience different levels of loneliness (11). With our large and robust sample, we are able to compare the DSH behavior in relation to loneliness for girls and boys separately. Also, few studies have examined the association of DSH with self-rated health before, even though DSH is seen as a serious health issue (6). This study hypothesised that (a) DSH is associated with loneliness and related factors, poor self-rated health and satisfaction with life and (b) girls and boys differ in these associations. The aims of this study are to examine the frequencies of DSH and loneliness among adolescents in the Northern Finland Birth Cohort 1986 (NFBC 1986) and whether DSH is associated with selected loneliness and related factors and if there exist differences in those factors among girls and boys.

## Materials and methods

### Population and procedure

The sample is based on a general population-based study – NFBC 1986, which was collected from the 2 northernmost provinces in Finland (Oulu and Lapland) comprising 9,432 live born infants (4,567 girls and 4,865 boys) whose expected date of birth fell between 1 July 1985 and 30 June 1986. They have been prospectively followed since the prenatal period with follow-ups at ages 7–8 years (1992–1994) and 15–16 years (2001–2002). The next data collection for the cohort members is slated for 2013, when they would be 27–28 years old.

When cohort members were 15–16 years old in 2001–2002, 9,340 of them were alive (99% of all), and the addresses of 9,215 were known. At that time, adolescents received a postal questionnaire concerning family structure, friends, school, health, living habits, hobbies and behavior (22). The study population consists of members of the NFBC 1986 who answered the question *I feel lonely* in the postal questionnaire in 2001–2002. Parents and adolescents who opposed to the use of their data (n=209) were excluded from the analysis. The study population included 7, 014 participants (3,641 girls and 3,373 boys, mean age: 15.5). The ethical committee of Northern Ostrobothnia Hospital District reviewed this study. Written informed consent was obtained from the parents and the adolescents in the follow-up study in 2001–2002.

### Variables used in the analysis

#### Dependent variable

We derived the item 18 *I harm or I would like to harm myself on purpose* 1 from the Youth Self-Report Scale (YSR) (23), which was part of the adolescents’ questionnaire. In this scale, the participants assessed whether each statement had been true for them during the preceding 6 months. The item was labelled as *deliberate self-harm or ideation* (DSH). The response alternatives for all YSR items were: 1, not true; 2, somewhat/sometimes true and 3, very true/often true. Those scoring 2 or 3 on item 18 were defined as having experienced DSH similar to the earlier study using the same scale (9).

#### Explanatory factors

Subjective experience of loneliness was measured by the single-item variable *I feel lonely from YSR*. The second YSR item was *I feel that no one likes me* and was recoded as Not being liked; No (response alternative 1) and Yes (2,3). We also chose the item *Do you have a close friend with whom you can confidentially discuss your matters?* The response alternatives being: 1, I have no close friends; 2, I have one; 3, I have two; and 4, I have several. These were recoded to number of close friends: one/more (response alternatives 2–4) and none (1).

Satisfaction with life was queried as *What is your opinion about your current life situation?* Response alternatives were: 1, I cannot say; 2, Very dissatisfied; 3, Fairly dissatisfied; 4, Fairly satisfied and 5, Very satisfied. These were recoded as Satisfied (response alternatives 4,5), Cannot say (1) and Dissatisfied (2,3). *Self-rated health* was assessed as *How would you describe your health?* The response alternatives were: 1, Very poor; 2, Poor; 3, Moderate; 4, Good and 5, Very good. These were recoded as Very good/good (response alternatives 4,5), Moderate (3) and Poor (1,2). Self-reported health status is a common variable for measuring the self-concept of health (14).

#### Statistical analyses

We used cross-tabulations to describe the frequency of the DSH by dimensions of loneliness, loneliness-related factors, self-rated health and satisfaction with life by gender. We employed logistic regression analyses to describe the association of DSH with all 5 selected explanatory factors with DSH. Wald's test was used to test the statistical significance of the OR where P values <0.05 were considered as statistically significant. IBM SPSS Statistics 19 was used to perform all statistical analyses.

## Results

A total of 608 adolescents (8.7%) reported DSH or ideation. More girls (n=488, 13.4%) than boys (n=120, 3.6%) reported DSH during the 6 months preceding the survey. In total, 221 adolescents (3.2%) of the respondents expressed that the statement *I feel lonely* was very true/often true; thus, they were very lonely. Girls reported it more frequently in comparison to boys (girls: n=149, 4.1%; boys: n=72, 2.2%). In total, 1850 respondents (26.4%) answered that the statement was somewhat/sometimes true; thus, they were experiencing some levels of loneliness and again girls reported loneliness more often than boys (girls: n=1,265, 34.8%; boys: n=585, 17.4%). Boys reported having fewer close friends than girls and there were more girls who felt that nobody liked them. There were no differences in self-reported health among boys and girls; most of them reported having very good or good health. Most adolescents were satisfied with their lives (see Table I).

**Table I:** Distribution of subjects according to loneliness, loneliness–related factors and self-rated health, prevalence of deliberate self-harm (DSH) during the past 6 months and logistic regression of DSH

	Girls							Boys						
		
		Reported DSH		Unadjusted		Adjusted^a^			Reported DSH		Unadjusted		Adjusted^a^	
								
Explanatory factor	No. of subjects	N	%	OR	95% CI	OR	95% CI	No. of subjects	N	%	OR	95% CI	OR	95% CI
Feeling lonely													
Never (ref.)	2,223	149	6.7					2,706	52	1.9				
Somewhat/sometimes	1,265	278	22.0	3.9	(3.1–4.8)	2.4	(1.9–3.1)	585	55	9.4	5.2	(3.8–7.8)	2.4	(1.5–3.9)
Very/often	149	61	40.9	9.6	(6.6–13.9)	4.1	(2.7–6.3)	72	13	18.1	11.2	(5.8–21.7)	3.2	(1.4–7.3)
Number of close friends													
One/more (ref.)	3,503	465	13.3					2,938	98	3.3				
None	122	22	18.0	1.4	(0.8–2.3)	0.5	(0.3–0.9)	360	19	5.3	1.6	(0.9–2.6)	0.6	(0.3–1.1)
Not being liked													
No (ref.)	2,320	167	7.2					2,781	42	1.5				
Yes	1,286	317	24.7	4.2	(3.4–5.1)	2.2	(1.8–2.9)	553	76	13.7	10.3	(7.0–15.3)	6.0	(3.8–9.5)
Self-rated health													
Very good/good (ref.)	2,994	312	10.4					2,862	79	2.8				
Moderate	590	162	27.5	3.2	(2.6–4.0)	2.0	(1.6–2.6)	458	37	8.1	3.0	(2.0–4.6)	1.7	(1.1–2.7)
Poor/very poor	46	14	30.4	3.7	(1.9–7.1)	1.7	(0.8–3.5)	32	4	12.5	5.0	(1.7–14.6)	1.4	(0.4–5.0)
Satisfaction with life													
Satisfied (ref.)	3,234	349	10.8					2,988	80	2.7				
Cannot say	167	52	31.1	3.7	(2.6–5.2)	2.6	(1.8–3.8)	133	12	9.0	3.6	(1.9–6.7)	1.8	(0.9–3.8)
Dissatisfied	210	85	40.5	5.6	(4.1–7.5)	3.3	(2.4–4.7)	182	28	15.4	6.6	(4.1–10.4)	3.3	(1.9–5.7)

a Adjusted for all factors in the table.

Among adolescents who reported DSH, 40.9% of girls and 18.1% of boys were very/often lonely and 22.0% of girls and 9.4% of boys were somewhat/sometimes lonely. Of those who reported DSH, 18.0% of girls and 5.3% of boys did not have any close friends, 24.7% of girls and 13.7% of boys felt that nobody liked them, 30.4% of girls and 12.5% of boys reported having poor health and 40.5% of girls and 15.4% of boys were dissatisfied with their lives.

Logistic regression was performed separately for girls and boys to assess the association of DSH with selected explanatory factors (see Table I). When controlling for all of the 5 variables in the model, the factors associated with having experienced DSH were feeling very/often lonely or somewhat/sometimes lonely (girls: OR 4.1 and 2.4, respectively; boys: OR 3.2 and 2.4, respectively), being dissatisfied with life (girls: OR 3.3; boys: OR 3.3), being unable to tell whether they were satisfied or not with their lives (only girls: OR 2.6), feeling of not being liked (girls: OR 2.2; boys: OR 6.0) and moderate self-rated health (girls: OR 2.0; boys: OR 1.7).

## Discussion

We studied the DSH or its ideation, feelings of loneliness, loneliness-related factors, self-rated health and satisfaction with life among of 16-year-old Finnish adolescents. The prevalence of DSH was 8.7% during the preceding 6 months, which was higher than prevalence found in reports from Australia and England (4,5). Very few earlier studies have examined DSH in Northern circumpolar areas. A Norwegian study, which used the same measurement as in this study, reported a prevalence of 12.5% among 487 adolescents aged 13–16 years. (9). In a Swedish longitudinal study, a 9-item Deliberate Self-Harm Inventory was employed to examine DSH prevalence among adolescents, and as many as 41.6% of the participants reported on the first measuring point that they had harmed themselves at least once in their lifetime and repeated self-harm (at least 5 instances) was reported by 18.3% of the adolescents (24). The large differences in the prevalence rates are likely to be due to the different methods, sample sizes and questionnaires used. The prevalence for DSH has been noted to be significantly higher for studies using anonymous methods, such as in this study, in comparison to non-anonymous methods (6). More generally, suicidal behavior is a severe public health concern in the Northern populations (25). Suicide rates are among the largest in the world, and suicides are one of the leading causes of death among youth (26), especially among indigenous young men (9). Girls reported DSH clearly more than boys (13.4% vs. 3.6%, respectively), similar to earlier reports (2,4,9). The 6-month prevalence of DSH was found to be low (2.2%) among adolescent Finnish boys by using the same measurement (18).

The prevalence of loneliness was 3.2%, and this result is in accordance with an earlier survey-based study conducted in Finland, which also used a single-item variable to assess loneliness and found that 3.6% of the youngest age group (age: 18–29 years) felt lonely (27). Girls reported loneliness more often than boys. This finding is similar to the findings of other Finnish studies (16,28) but contrary to those published in some other countries, such as Canada or UK (29,30). However, these studies are not directly comparable to ours because of the differences in the measurement of loneliness. There was a significant association between DSH and loneliness among girls and boys. However, similar to other studies, an association between the number of close friends and DSH was not found (31). One explanation for the association between DSH and loneliness and the higher prevalence of DSH among girls and the higher prevalence of suicide among boys can be found from the varied and changing social relations during childhood and adolescence. Even though gendered patterns in social relations are changing (32), there seems to be a tendency wherein from early on girls are still taught to be kind and friendly to other people, to avoid conflicts (33) and to be caring, more so than boys. This can be interpreted girls to maintain the behavior of girls to think, worry and invest in social relations more than boys (32). Same-sex friendships among girls are generally considered more emotionally expressive and intimate than boys; girls spend time in smaller groups, while friendship among boys are considered more activity-oriented and consist of larger groups (32,34). Boys are often encouraged to be “tough”, self-expressive and more independent than girls. The spectrum of allowed emotions for boys might be stricter than for girls; therefore, it might not be as socially acceptable for them, as it is to girls, to express different emotions (35). Therefore, boys might not report different emotions or feelings as often in surveys or in general, as girls, and emotional and social difficulties might cause more emotional distress among boys. Loneliness can be very distressing for both girls and boys (11), but girls may have evolved better coping strategies to deal with emotional distress than boys. According to Gilligan and Machoian (36), DSH can be seen as a form of communication. Some girls may learn that when they threaten or actually harm themselves, their distress is heard (3,36). The suicidal and DSH behavior of girls includes signs of hope related to their social relationships; therefore, they turn to DSH behavior rather than actually committing suicide (36). Boys might notice the difficult emotions at a later stage, when the situation is already hopeless, and in serious cases, instead of turning to self-harming behavior, they turn to more violent methods (6); boys commit suicide much more often than girls.

Furthermore, feelings of not being liked were significantly associated with DSH in both girls and boys. The OR was higher among boys than girls, suggesting that it is important to notice that adolescent girls and boys need trustworthy and supportive people around them. Without this, they have a higher risk of DSH.

After adjusting for other factors in the regression model, DSH was significantly associated with moderate self-rated health among girls and boys, but not with poor health, which may result from intermediating effect of life satisfaction between poor health and DSH. However, this could be explained by the small number of subjects answering that their health was poor or very poor. DSH was clearly associated with dissatisfaction with life for girls and boys; obviously, those involved in DSH were not necessarily satisfied with their lives, and the motives for DSH may be to express their desperation (7).

Finally, the experiences of loneliness and DSH can be context-related (37,38). The members of NFBC 1986 experienced a severe economic recession in Finland at the beginning of the 1990s that involved a reduction in state funding for health, social and educational services. The economy revived in the mid-1990s, but recession in public sector services continued (39). In Particular, in northern Finland, the issues of urbanisation and “death” of small villages, long distances and other social and economic problems, such as the poor availability of educational facilities and job opportunities (40), still exist. As an on-going problem, they might contribute to growing marginalisation, loneliness, malaise and possibly to DSH and suicides among northern Finnish adolescents, and other Northern communities might face similar challenges.

The strength of this study is the large general population birth cohort and a sample size that consists of more than 7,000 adolescents. The main limitations of the study are that the motives behind the DSH acts or the methods used for harming oneself were not researched, and the wording of the YSR item was changed. Therefore, the results are not exactly comparable to other studies that reported different methods behind the DSH behavior or used the YSR scale. Furthermore, cross-sectional study design prevents us from claiming the causality of the observed associations.

## Conclusion

An association between DSH and experiences of loneliness were found. Loneliness and DSH should be viewed as serious social and health problems, since they have many negative consequences for an individual's well-being and, in some cases, may lead to suicide. Parents might be unaware of their children's DSH behavior (9). Identifying the lonely adolescent might be difficult due to the subjective nature of the experience. Health providers, parents and teachers should gain more knowledge about DSH and related factors, such as loneliness, and be updated on measures and tools for identifying the adolescent with these behaviors (41) in order to help them. Furthermore, the adolescents themselves should be encouraged to seek help (1), and more resources are required for student counselling. A whole-school approach, including the parents, aimed at enhancing emotional and social well-being could be helpful for changing the ethos and culture of the school. Belonging, non-violence, awareness, openness and confidence in dealing with emotions and sensitive issues should be emphasised (8) in schools and at home. Children and adolescents should be taught emotional intelligence and empathy, which could help them discuss their emotions and feelings more openly without the fear of social stigma in a safe environment.

Finally, DSH is common among adolescent girls. Suicide rates among girls are increasing, yet the malaise and suicidal behavior of girls has been underexamined. Future research using longitudinal and mixed methods research design should be conducted to gauge the possible stability and causality of DSH experiences and their relationship to loneliness and gender more thoroughly.

## Footnotes

1 This is the wording of the item as stated in Finnish. The English translation in the questionnaire was: ‘I deliberately try to hurt or kill myself’. In the Finnish version, the question was changed by its wording, since it was thought that if the statement has the word suicide in it, it might give adolescents ideas of suicide.
